# “Breaking the Bleed” Spontaneous Hemorrhage in an Emphysematous Bulla Complicated by Anticoagulation: A Case Report

**DOI:** 10.7759/cureus.76574

**Published:** 2024-12-29

**Authors:** Fahad H Shaikh, Nicholas A Zerilli, Taimoor Jamil, Daniyal Ishtiaq, Deepti Avasthi

**Affiliations:** 1 Internal Medicine, Mercy Health St. Vincent Medical Center, Toledo, USA; 2 Internal Medicine, Mercy Health, Toledo, USA; 3 Internal Medicine, Bon Secours Mercy Health, Toledo, USA

**Keywords:** chronic obstructive pulmonary disease (copd), pulmonary bulla, pulmonary hemorrhage, therapeutic anticoagulation, video-assisted thoracoscopic surgery (vats)

## Abstract

We present a case of spontaneous hemorrhage in an emphysematous bulla, complicated by anticoagulation. Bullous emphysema is a well-recognized complication of chronic obstructive pulmonary disease (COPD), and a rare manifestation is hemorrhage into preexisting pulmonary bullae. A 69-year-old male patient presented to the emergency department with hemoptysis, shortness of breath, and productive cough. He had a history of COPD, coronary artery disease, congestive heart failure, and paroxysmal atrial fibrillation, managed with aspirin and rivaroxaban. Chest imaging revealed fluid-filled bulla in the left lower lobe, suggesting spontaneous hemorrhage into an emphysematous bulla. Bronchoscopy confirmed active bleeding, and the patient was managed with medical therapy and video-assisted thoracoscopic surgery with bullectomy. The patient recovered well post-operatively, with no further episodes of bleeding. Long-term follow-up showed improved respiratory function and the successful placement of a left atrial appendage closure device to manage atrial fibrillation to obviate the need for long-term anticoagulation. In this report, we will discuss prior cases of this condition and its management.

This case not only sheds light on the rare and complex clinical scenario of a spontaneous bleed in a pre-existing emphysematous bulla but also underscores the successful use of surgical resection in a context where surgical management is not often used. Our patient’s positive outcome and recovery over seven months, as evidenced by close longitudinal follow-up, provides valuable insights into the potential benefits of surgical intervention in select cases.

## Introduction

Pulmonary bullae, especially in patients with chronic obstructive pulmonary disease (COPD), are air-filled spaces within the lungs caused by the destruction of alveolar walls. Pulmonary bullae are present in around 5% of the general population and nearly 12% of adults over the age of 30 globally. Bullae can be particularly dangerous when they become large, as they may cause dyspnea, spontaneous pneumothorax, or, in very rare cases, hemorrhage [1]. Although complications such as pneumothorax are well documented, spontaneous bleeding into bullae is an exceedingly rare phenomenon, seen more commonly in patients receiving anticoagulant or antiplatelet therapy [2]. This report presents the case of spontaneous hemorrhage within an emphysematous bulla, complicated by the patient's use of antiplatelet and anticoagulant medications for coronary artery disease (CAD) and atrial fibrillation.

## Case presentation

A 69-year-old man presented to the emergency department due to concern of hemoptysis on the morning of presentation. He is known to have GOLD (Global Initiative for Chronic Obstructive Lung Disease) class IV COPD with severe emphysema and bullous disease, chronic respiratory failure on 2 liters of home oxygen via nasal cannula, former smoker with a 70 pack-year smoking history, CAD with a right coronary artery drug-eluting stent, paroxysmal atrial fibrillation, and congestive heart failure (CHF) with reduced ejection fraction of 40-45%. Of note, the patient had been using his home medications aspirin 81 mg and rivaroxaban 20 mg for his aforementioned medical conditions. Other home medications include amiodarone 200 mg, albuterol inhaler, furosemide 20 mg, losartan 50 mg, and roflumilast 250 mcg.

The patient awakened on the morning of the day of presentation coughing up both fresh blood and clots. He estimated that he had produced roughly half a cup of both blood and clots that morning. Over the past week, the patient had increased shortness of breath and a productive cough with phlegm.

In the emergency department, the patient was afebrile with a temperature of 36.5°C and hemodynamically stable with a blood pressure of 122/60 mm Hg. Complete blood count showed a hemoglobin level of 12.3 g/dL and leukocyte count of 9,700 cells/uL. C-reactive protein was elevated at 6.3 mg/dL and procalcitonin was elevated at 0.40 ng/mL, findings concerning for bacterial infection. He did not require blood transfusion. The patient’s prothrombin time was 15.1 seconds, and the international normalized ratio (INR) was 1.2. The patient was placed on 2 liters of nasal cannula oxygen, which is his baseline oxygen requirement at home, and this was eventually titrated up to 5 liters.

**Figure 1:**
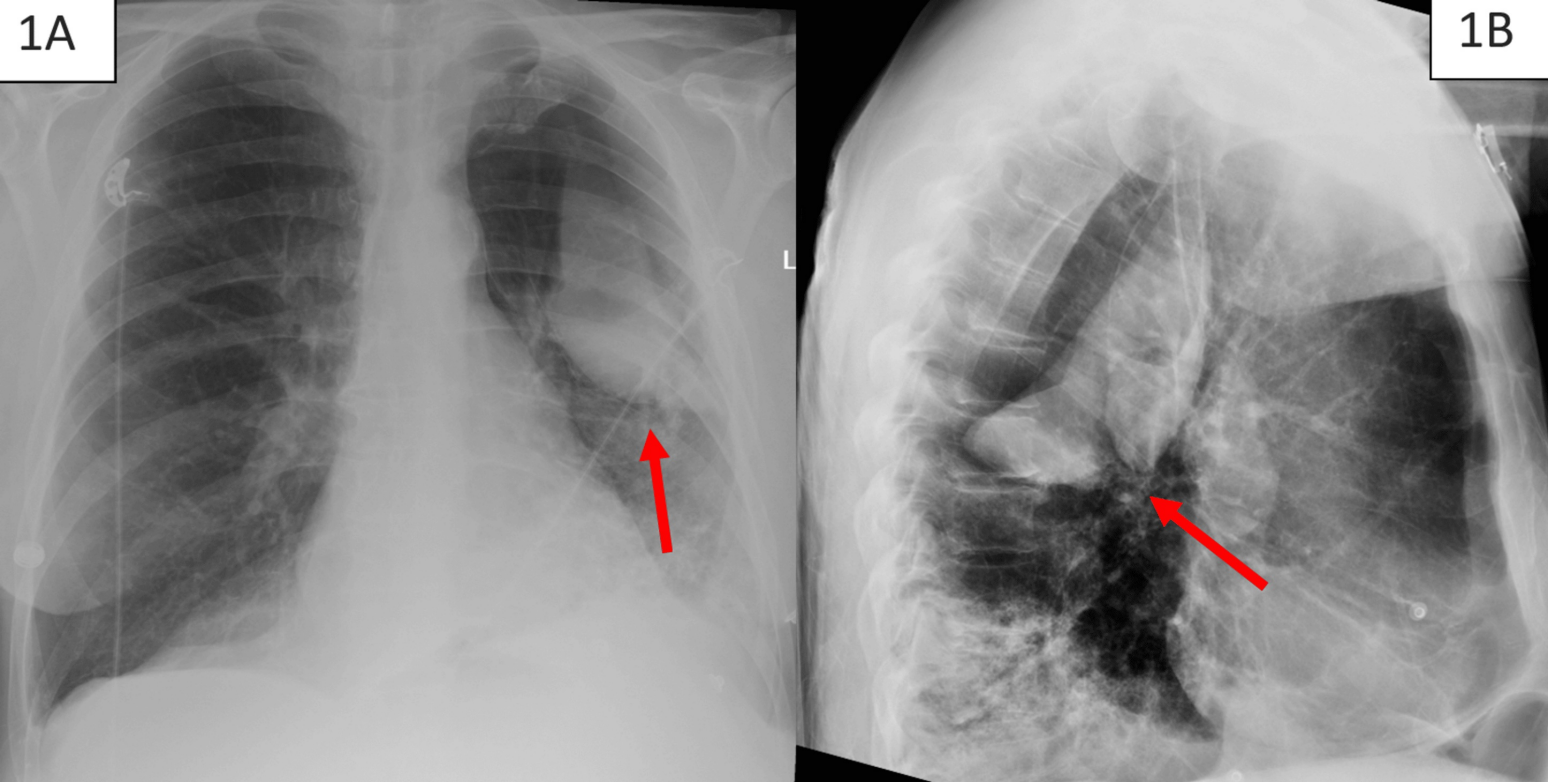
(A) Posteroanterior view and (B) lateral view of the chest X-ray showing fluid-filled bulla (arrow) in the superior segment of the left lower lobe of the lung Extensive consolidation in the left lower lobe predominately affecting the posterior basal segment, with small left pleural effusion, was also noted.

**Figure 2:**
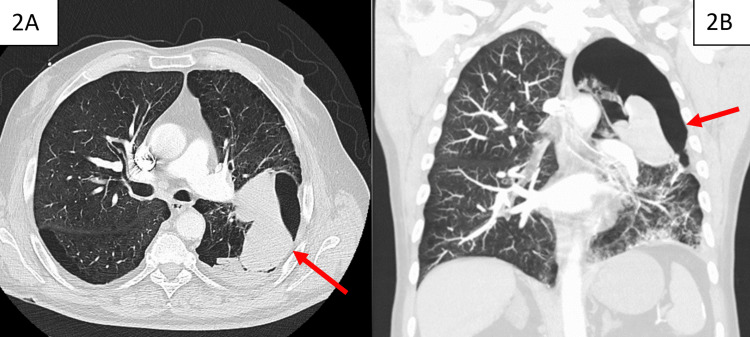
(A) Axial view and (B) coronal view of the chest CT showing a large hemorrhagic bulla (arrow) in the superior segment of the inferior left lobe

**Figure 3:**
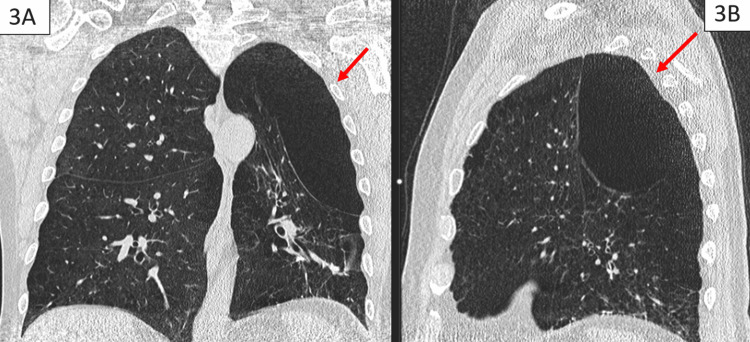
(A) Sagittal view and (B) axial view of the low-dose CT of the chest one month prior to presentation showing a large left-sided bulla without hemorrhage CT of the chest with bronchial artery shown (arrow) to not supply the pulmonary hemorrhage.

An initial chest X-ray was obtained (Figure 1), which demonstrated fluid opacifying a large bulla in the superior segment of the left lung lower lobe and extensive consolidation in the left lower lobe predominately affecting the posterior basal segment, with small left pleural effusion. A follow-up CT pulmonary angiography was ordered and performed on the same day (Figure 2). This CT chest with pulmonary angiography was compared to the previous low-dose CT conducted outpatient for lung cancer screening (Figure 3) and demonstrated consolidative opacity with an air-fluid level in the left lower lobe with intermediate-density material filling a previously demonstrated left lower lobe bulla, 9.0 cm in size at the largest diameter per radiology. This consolidative opacity was deemed to be a spontaneous pulmonary hemorrhage filling an emphysematous bulla. The patient was admitted to the intensive care unit for close monitoring of his respiratory status.

In the intensive care unit, pulmonology conducted a fiberoptic bronchoscopy. During bronchoscopy, the posterior basal segment of the left lower lobe posterior bronchus (LB 10) was noted to be erythematous and to have fresh blood emanating out of this segment. Bronchial alveolar lavage was conducted at that time. Subsequent cultures, which have been finalized, showed only normal respiratory flora including *Neisseria catarrhalis*, diphtheroids, alpha-hemolytic streptococci, and *Candida albicans*, with which the patient has had known colonization. Regardless, the patient was treated with piperacillin-tazobactam via intravenous access during his hospital stay, given his increased phlegm production and concern for an underlying infection. Hemoptysis decreased but still continued episodically despite holding anticoagulation and antiplatelet agents. He received IV steroids methyl-prednisone 40 mg thrice daily. No procoagulants were administered.

**Figure 4:**
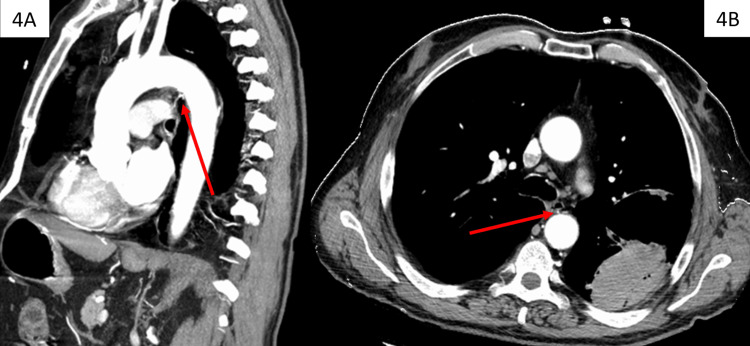
(A) Sagittal view and (B) axial view of chest CT with bronchial artery (arrow) shown to not supply the pulmonary hemorrhage

A chest CT angiogram with IV contrast (Figure 4) was conducted to determine if the patient was a candidate for bronchial artery embolization via interventional radiology under fluoroscopy.

After completion, the patient was deemed to not have active extravasation of contrast dye within the large left lower lobe bulla and was not a candidate for bronchial artery embolization per the consulting interventional radiologist.

With continued hemoptysis after initial conservative management for infection and stopping anticoagulation and antiplatelets, after the patient was deemed not to be a candidate for embolization by interventional radiology, and due to compression of the left upper lobe from the large hemorrhagic bullae, thoracic surgery was consulted for consideration of bullectomy on day 9 of hospitalization. The patient was deemed a candidate for video-assisted thoracoscopic surgery (VATS) with bullectomy. Arterial blood gas analysis preoperatively showed pH 7.31, pCO_2_ 55.3 mmHg, and HCO_3_ 27.5 mEq/L, evidence of chronic respiratory acidosis secondary to COPD. During VATS, four 10-mm ports were established on the left chest wall: two ports in the 7th intercostal space and two ports in the 11th intercostal space. Once the surgeons visualized the thoracic cavity, adhesions were noted around the large lower lobe bulla. Adhesions were lysed. Subsequently, the large hemorrhagic bulla margins were clarified. Using 60 staples, the bulla was isolated and stapled off from lung parenchyma. A specimen retrieval pouch was used to retrieve the resected bulla. Platelets and air leak sealant were applied to the resection margins of the lung. Two separate 36-Fr chest tubes were placed in the seventh intercostal space. At the closure of the procedure, both chest tubes were placed for suction.

On postoperative day 2, chest tubes were placed to water seal. Both chest tubes were subsequently removed successfully without air leak or pneumothorax noted clinically or on radiography. Throughout the patient’s admission, antiplatelet and anticoagulation therapy was held. However, the patient did receive deep venous thrombosis prophylaxis with enoxaparin 40 mg daily. Pathologic analysis of the bulla sample showed "the pleura is focally ragged and sectioning reveals dilated air-filled spaces, many of which are disrupted and measure up to 7.0 cm. There are no obvious masses. The remaining lung parenchyma displays gross emphysematous change."

On day 18 of admission, the patient was discharged in a medically stable condition. During hospitalization, he had completed an initial course of intravenous piperacillin-tazobactam for 14 days, followed by switching to oral amoxicillin-clavulanate. At this time, the patient was on his home level of oxygen of 2 liters. He was no longer experiencing hemoptysis. Given the concern of rebleeding and with consultation and expert opinion from pulmonology and cardiothoracic surgery, anticoagulation was held. The patient had left atrial appendage occlusion performed by interventional cardiology shortly after discharge. Antiplatelet agents were continued, and the patient was able to take dual antiplatelet therapy after the left atrial occlusion procedure. The patient was also discharged with a prescription for amoxicillin-clavulanate (875 mg-125 mg) twice daily by mouth. Follow-up was recommended with thoracic surgery in one to two weeks, cardiology in three weeks, pulmonology in one week, infectious disease in one month, and his primary care provider in one week.

Subsequent follow-up with the thoracic surgeon was reassuring, with the patient no longer experiencing hemoptysis and his shortness of breath improving from hospital admission. The patient followed up with cardiology in the outpatient setting and successfully underwent left atrial appendage device placement. It should be noted that after the placement of the left atrial appendage device, the patient was prescribed dual antiplatelet therapy with aspirin 81 mg daily and clopidogrel 75 mg daily. This regimen is to be continued for six months after device placement, and the patient will thereafter be on antiplatelet monotherapy with aspirin 81 mg daily. This therapy was discussed with and approved by the patient’s primary pulmonologist. The patient was thoroughly educated on the risk of hemoptysis and was told to seek medical attention immediately if he experiences such.

**Figure 5:**
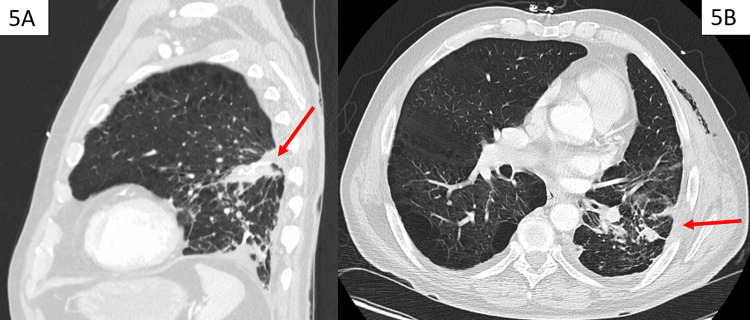
(A) Sagittal view and (B) axial view of chest CT one month after hospital discharge showing post-surgical fibrosis of lung parenchyma and pleura

Upon following up with his pulmonologist, the patient was continued on home oxygen of 2 liters, levalbuterol 45 mcg with one puff inhaled every 4 hours for wheezing/shortness of breath, roflumilast 250 mcg tablet daily, and triple therapy daily inhaler with contents of fluticasone 200 mcg/umeclidinium 62.5 mcg/25 mcg vilanterol. Subsequent chest computed tomography (CT) showed successful surgical resection of hemorrhagic bullae with corresponding fibrosis (Figure 5). He was also referred to pulmonary rehabilitation by a pulmonologist and has since completed pulmonary rehabilitation. His oxygen requirement was reassessed to be intermittent with significant exertion and overnight, demonstrating improvement from his previous oxygen requirement.

## Discussion

In patients with COPD, especially the emphysematous subtype, bullae are a common complication. A bulla is described as an air space in the lungs measuring greater than 1 cm [3] and can be so large as to occupy over 30% of the lung, so-called “giant bullae." Pulmonary bullae are present in around 5% of the general population and nearly 12% of adults over the age of 30 years globally [4]. Bullae are characterized by their thin walls (typically less than 1 mm) and their subpleural location. Functionally, they are classified as areas of minimal gas exchange, i.e., “dead space," in part due to the destruction of the normal vascular architecture of the alveoli.

Despite over half a century of research on emphysematous bullae, our understanding of the disease's nature, its complications, and the optimal management strategies remains incomplete. Most complications of emphysematous bullae are generally rare, including progressive hypoxia and spontaneous pneumothorax. Treatment strategies generally tend to be conservative. Bullectomy is generally reserved for patients with significant dyspnea, giant bullae, and/or recurrent pneumothoraxes [5].

Interestingly, because of the damaged architecture of the bulla tissue, including alveolar destruction, fibrosis, and reduced vasculature, spontaneous bleeding from a bulla is extremely rare. In a notable case report by Mouly et al. [6], a patient with Wegener's granulomatosis and coexisting giant tumors experienced severe pulmonary hemorrhage without any bleeding inside the tumor itself, further underscoring the rarity of such complications. We reviewed 16 reported cases of spontaneous lung hematoma, of which only four involved spontaneous bleeding within a pre-existing emphysematous bulla, as in our reported case. Other cases were associated with parenchymal bleeding, infections, secondary causes of vasculitis, or cystic lung diseases. These studies also identify risk factors that likely increase the risk of hemorrhage into emphysematous bullae, which include infections, COVID-19-related vasculitis, the presence of lung tumors, or COPD exacerbations [7,8]. These may lead to hemorrhage via destabilizing the bullous walls or causing bleeding from pulmonary or pleural vessels that present as bleeding into bullae. One of the most consistent risk factors across all reported cases is anticoagulation or antiplatelet medication, present in nearly all cases.

Our case presented with a large bleed into a pre-existing emphysematous bulla, complicated by a history of CAD on antiplatelet therapy, atrial fibrillation on anticoagulant therapy, CHF, and stage IV COPD with chronic hypoxic respiratory failure on home oxygen. Given the limited availability of guidelines, we initiated an empiric treatment protocol, which included holding anticoagulation and antiplatelet therapy, preparing to administer blood products to the patient as needed, and initiating broad-spectrum antibiotics to cover for a potential occult infection that could trigger bleeding through infection-related angionecrosis. This approach was largely consistent with treatment strategies described in the other reported cases.

Further interventions included bronchoscopy to assess for an active source of bleeding and a CT-guided angiogram to identify any potential feeding vessels. Embolization under fluoroscopic guidance was considered but ultimately not pursued, as the patient had some improvement of hemoptysis with conservative treatment, and no bleeding vessel was identified on imaging. Additionally, there is a lack of evidence on the risks and benefits of embolization in this context. This is an avenue for further study of patients who are candidates and could benefit from embolization.

After thorough discussion about the risks of rebleeding and thrombotic complications between a multidisciplinary team consisting of cardiology, pulmonology, and cardiothoracic surgery, the patient was offered surgical bullectomy. The patient had an uneventful intraoperative and postoperative period, showing significant recovery, and was eventually discharged home on oral anticoagulation.

This case had striking similarities to other case reports, where all patients were male, on anticoagulation, and experienced near-fatal bleeds without identifiable bleeding vessels. All were also smokers, and none was on supratherapeutic doses of anticoagulation. Interestingly, despite the lack of overt infection, all cases were treated empirically with antibiotics. Our case diverged in the approach taken after the resolution of the initial bleed. Instead of conservatively managing the bulla, as is typical, we proceeded with surgical resection. Prior cases mentioned considering surgical or radiological intervention but were largely managed conservatively, mentioning hemodynamic stability as a factor contributing to the decision to not pursue surgical intervention. This case therefore demonstrates that surgery- or radiology-guided interventions may have an increasing role in the management of this rare condition moving forward. Bullectomy allowed us to safely restart the patient's life-saving antiplatelet therapy, minimizing the risk of rebleeding while addressing the potential cardiac complications. Additionally, our patient demonstrated significant improvement in quality of life, oxygenation, and physical endurance, as evidenced by improvements in the 6-minute walk test and reduced oxygen requirements following pulmonary rehabilitation sessions.

## Conclusions

This case report describes a rare and complex clinical scenario of a spontaneous bleed in a pre-existing emphysematous bulla, as well as the interdisciplinary management of a condition that does not have specific guidelines at this time. It also underscores the successful use of surgical resection in a context where surgical management is not part of the usual treatment. The case provides a useful framework for clinicians to reference in similar situations and a stepping stone for further guidelines from relevant societies. Our patient’s positive outcome and recovery over seven months, as evidenced by close longitudinal follow-up, provides valuable insights into the potential benefits of surgical intervention in select cases. This case highlights the importance of further research into identifying those at higher risk for such rare complications and understanding the long-term outcomes of different treatment strategies in emphysematous bullous disease, including surgical and interventional radiology-guided treatment options. Further research is required in this space to delineate case characteristics and outcomes using these techniques.
